# REIMAGINING WORK: TAPPING INTO THE STRENGTHS OF OLDER ADULTS

**DOI:** 10.1093/geroni/igae098.0408

**Published:** 2024-12-31

**Authors:** Kathryn Nearing, Katy Terveer, Ronica Rooks

**Affiliations:** Chair; Co-Chair; Discussant

## Abstract

This session will focus on inspiring and informing the audience about how we can reimagine the way we work in order to benefit from the experience, skills, and wisdom of older workers. We will explore the modern trends that are enabling us to work longer and the history of ageism in the workplace. We will help aging advocates to understand and promote the benefits of working longer from an individual, employer, and societal level. Finally, we will look at some of the strategies that organizations can leverage in order to recruit and retain older workers as well as leverage the full potential of an age-diverse workforce.

